# From Nonspecific DNA–Protein Encounter Complexes to the Prediction of DNA–Protein Interactions

**DOI:** 10.1371/journal.pcbi.1000341

**Published:** 2009-04-03

**Authors:** Mu Gao, Jeffrey Skolnick

**Affiliations:** Center for the Study of Systems Biology, School of Biology, Georgia Institute of Technology, Atlanta, Georgia, United States of America; University of Kansas, United States of America

## Abstract

DNA–protein interactions are involved in many essential biological activities. Because there is no simple mapping code between DNA base pairs and protein amino acids, the prediction of DNA–protein interactions is a challenging problem. Here, we present a novel computational approach for predicting DNA-binding protein residues and DNA–protein interaction modes without knowing its specific DNA target sequence. Given the structure of a DNA-binding protein, the method first generates an ensemble of complex structures obtained by rigid-body docking with a nonspecific canonical B-DNA. Representative models are subsequently selected through clustering and ranking by their DNA–protein interfacial energy. Analysis of these encounter complex models suggests that the recognition sites for specific DNA binding are usually favorable interaction sites for the nonspecific DNA probe and that nonspecific DNA–protein interaction modes exhibit some similarity to specific DNA–protein binding modes. Although the method requires as input the knowledge that the protein binds DNA, in benchmark tests, it achieves better performance in identifying DNA-binding sites than three previously established methods, which are based on sophisticated machine-learning techniques. We further apply our method to protein structures predicted through modeling and demonstrate that our method performs satisfactorily on protein models whose root-mean-square Cα deviation from native is up to 5 Å from their native structures. This study provides valuable structural insights into how a specific DNA-binding protein interacts with a nonspecific DNA sequence. The similarity between the specific DNA–protein interaction mode and nonspecific interaction modes may reflect an important sampling step in search of its specific DNA targets by a DNA-binding protein.

## Author Summary

Many essential biological activities require interactions between DNA and proteins. These proteins usually use certain amino acids, called DNA-binding sites, to recognize their specific DNA targets. To facilitate the search of its specific DNA targets, a DNA-binding protein often associates with nonspecific DNA and then diffuses along the DNA. Due to the weak interactions between nonspecific DNA and the protein, structural characterization of nonspecific DNA–protein complexes is experimentally challenging. This paper describes a computational modeling study on nonspecific DNA–protein complexes and comparative analysis with respect to specific DNA–protein complexes. The study found that the specific DNA-binding sites on a protein are typically favorable for nonspecific DNA and that nonspecific and specific DNA–protein interaction modes are quite similar. This similarity may reflect an important sampling step in the search for the specific DNA target sequence by a DNA-binding protein. On the basis of these observations, a novel method was proposed for predicting DNA-binding sites and binding modes of a DNA-binding protein without knowing its specific DNA target sequence. Ultimately, the combination of this method and protein structure prediction may lead the way to high throughput modeling of DNA–protein interactions.

## Introduction

DNA-binding proteins play an essential role in many fundamental biological activities, including DNA transcription, replication, packaging, repair and rearrangement. Interactions relevant to these activities typically involve specific binding sites on both proteins and DNA. Over the past several decades, many efforts have been made in order to understand basic principles that determine the specific DNA-protein interactions. It is well-known that there does not exist a simple recognition code between protein amino acids and DNA base pairs [1]–[4]. This poses a great challenge for the prediction of DNA-protein interactions.

The daunting task of elucidating DNA-protein interactions can be addressed with the assistance of computational modeling. Methods for docking the complex from separated protein/DNA structures have been developed [5]–[7]. As an early example, the Monte Carlo program MONTY has been applied to sample configurations of a single DNA-protein complex in the vicinity of its native state [6]. The development of an efficient geometric recognition algorithm [8], which allows a global search for optimal surface complementarity though rigid body rotation and translation, greatly advanced the molecular docking field. An implementation of the algorithm, FTDOCK, was applied to DNA-protein docking [5], with encouraging benchmark results reported on modeling eight DNA/repressor complexes starting from unbound protein structures and canonical B-DNA. A more recent approach, HADDOCK, starts with a similar rigid body docking procedure, followed by semi-flexible refinement [7]. Excellent docking models were obtained for three examples by HADDOCK.

The docking methods assume the availability of both protein and DNA structures. Given only the structure of a DNA-binding protein, it is of interest to determine the DNA-binding protein residues without the knowledge of the associated specific DNA sequence and structure with which the protein interacts. In the last few years, several methods have been developed to address this problem [9]–[15]. Most focus on analyzing characteristic patterns of DNA-binding residues from the solved structures of complexes. Standard machine-learning techniques, such as Support Vector Machine [10],[13] and neural networks [9],[14], have been adopted to differentiate DNA-binding residues from non-DNA-binding residues, using features like sequence composition, evolutionary profile, solvent accessibility, and electrostatic potential. Recently, a knowledge-based method DBD-Hunter that combines structural comparison and evaluation of a statistical pair potential was proposed for predicting DNA-binding proteins and associated binding residues [11]. The method yields an accuracy of 87% on DNA-binding site prediction in comprehensive benchmarks. However, the method is limited by the availability of appropriate DNA-protein complex structures to be used as templates.

In this study, we present a novel approach for predicting the protein residues that bind DNA and DNA-protein interaction modes, given the structure of a DNA-binding protein as the input. We systematically docked 44 specific DNA-binding proteins in both holo (DNA-bound) and apo (DNA-free) forms to a nonspecific canonical B-DNA molecule. Using energy evaluation and model clustering, we obtained representative complex models that provide structural insights into how DNA-binding proteins interact with a nonspecific DNA sequence. For about 80% of the proteins, the sites for specific DNA recognition are among the favorable interaction sites for nonspecific DNA binding. Furthermore, the interaction modes observed in the top ranked, nonspecific DNA-protein encounter complexes bear a certain similarity to the specific DNA-protein binding mode in the experimental structure. The biological implications of this similarity are discussed. Moreover, we demonstrate that our approach achieves better performance than three established methods based on machine-learning techniques. In addition to experimental structures, we show that our method can be applied to predicted protein models, generated by the state-of-the-art modeling program TASSER [16]. Satisfactory results were obtained for protein models with a root-mean square deviation, RMSD, ≤5 Å of their Cα atoms from their native holo-structures. We also show that our method can be further improved by considering conformational changes of DNA.

## Results

### DNA-Binding Site

**Figure 1:**
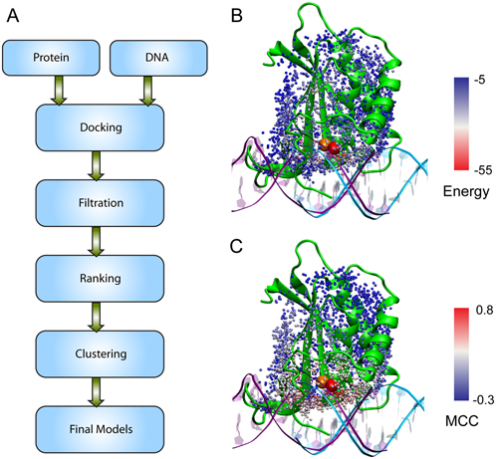
Methodology overview. (A) Flowchart of the DNA-protein complex modeling process. (B–C) An example, Epstein-Barr nuclear antigen-1, illustrates that specific-DNA recognition sites on a DNA-binding protein are energetically favorable interaction sites for nonspecific DNA. A nonspecific DNA (cyan) composed of 16 dA·dT base pairs was docked to the protein structure (green), which is complexed with a specific DNA molecule (purple) in the native structure (Protein Data Bank (PDB) code 1b3t). Each point represents one of top 2500 energy-ranked docking models. They are placed at the center of mass (COM) of the interfacial protein residues for a given docked pose, and are color scaled according to (B) energy values and (C) Matthews correlation coefficients. The spheres mark the location of the COM of DNA-interaction sites for the top model (red) and the native structure (orange).

The apo- and holo-structures of 44 non-redundant specific DNA-binding proteins (Table S1) are docked separately to a nonspecific B-DNA composed of 16 dA·dT base pairs, following the modeling procedure illustrated in Figure 1A. For each structure, we keep the top 2500 docking complex models ranked by their DNA-protein interfacial energy. We first compare DNA-interacting protein residues observed in top ranked encounter complexes with those observed in the native (experimental) complex structures. For this purpose, the Matthews Correlation Coefficient (MCC) is used to quantify the similarity between interaction sites for specific and nonspecific DNA on the protein's surface. A complex model is considered near-native if the associated MCC is higher than 0.5, which is the mid-point between perfect overlap (MCC = 1.0) and a random model (MCC = 0.0). As a representative example, Figure 1B and 1C show the energy and MCC for the top 2500 docked structures of Epstein-Barr nuclear antigen-1, whose top energy ranked model is a near-native model with a high MCC of 0.76.

**Figure 2:**
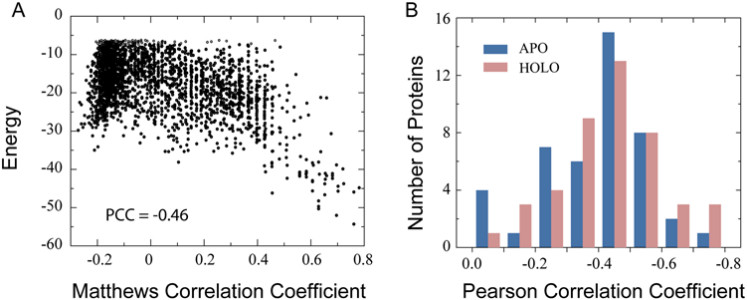
Correlation between MCC and DNA-protein interfacial energy. (A) A representative MCC versus energy plot shows the top 2500 docking solutions of Epstein-Barr nuclear antigen-1, as shown in Figure 1B and 1C. The correlation between MCC and energy was measured by Pearson correlation coefficient. (B) The histograms of PCCs of DNA-binding proteins from APO/HOLO sets.

Analysis of docking solutions suggests that specific DNA-binding sites on proteins are typically among the energetically favorable sites for sampling the nonspecific DNA. As shown in Figure 2, the MCC between specific and nonspecific DNA-binding sites is anti-correlated with the DNA-protein interfacial energy. A representative example is provided for the Epstein-Barr nuclear antigen-1, which has a Pearson Correlation Coefficient (PCC) of −0.46 between MCC and the interfacial energy (Figure 2A). On average, the PCCs are −0.40/−0.43 for the APO/HOLO sets, respectively (Figure 2B). Although the correlation is not very strong, the analysis does indicate that the specific DNA-binding sites on the protein are more likely involved in forming encounter complexes with a nonspecific DNA, as compared to the other regions of the protein. These nonspecific encounter complexes provide a structural basis for understanding the process known as facilitated diffusion [17],[18], during which a DNA-binding protein diffuses along nonspecific DNA in search of its specific DNA target sequence (see Discussion). For the purpose of sampling DNA sequence, the DNA-binding sites on the protein surface are energetically favorable to both specific and nonspecific DNA, resulting in the observed overlap between these sites.

**Figure 3:**
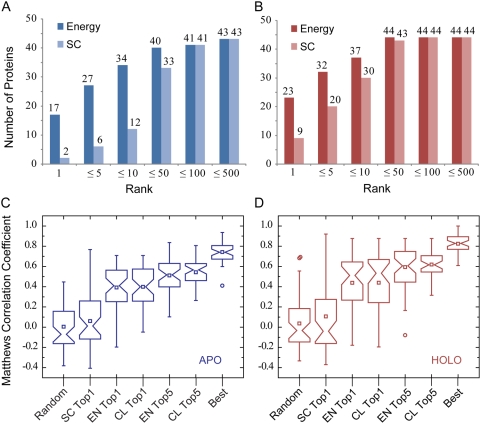
Specific DNA-binding sites *versus* nonspecific DNA-interacting sites observed in complex models. Models were built with apo (blue) and holo (red) protein structures. (A,B) Histograms of structures with at least one near-native model at different ranks. The models were ranked according to their interfacial energy or shape complementarity score. (C,D). Each box plot represents the MCCs of DNA-binding protein residue prediction in the APO/HOLO sets. The MCCs were calculated based on models selected from 2500 docking solutions under seven different model selection schemes. The lower, middle and upper quartiles of each box are the 25th, 50th, and 75th percentile; whiskers extend to a distance of up to 1.5 times the interquartile range. Outliers and means are represented by circles and squares, respectively. SC, EN, and CL denote ranking schemes using shape complementarity, energy, and clustering. Top1 and Top5 designate the top model and the best of top five models, respectively. The same notation is adopted throughout this paper.

One can utilize this observation to predict specific DNA-binding sites on protein through analyzing nonspecific DNA-protein docking solutions. Figure 3A and 3B show the number of proteins with at least one near-native complex model under various rank thresholds. According to the DNA-protein interfacial energy, we obtained a near-native top one model for 17 (39%) and 23 (52%) proteins, using apo and holo protein structures for docking, respectively. By comparison, shape complementarity ranking merely provides 2 (5%) and 9 (20%) proteins with a near-native top one ranked model based on apo- and holo-structures. Among the top ten energy ranked models, one can find at least one near-native model for 34 (77%) and 37 (84%) proteins from the APO and HOLO sets, while only 12 (27%) and 30 (68%) proteins from the same sets have a near-native model on the top ten list based on shape complementarity ranking.

To further improve model selection, we introduced a clustering procedure and compared various model selection schemes shown in Figure 3C and 3D. As expected, a randomly chosen model from the 2500 docking solutions gives a mean MCC very close to zero, 0.005/0.036 on the APO/HOLO sets, respectively. The mean MCC values of the top one shape complementarity ranked models, 0.06/0.11 on APO/HOLO sets, are slightly better than the means of random models. A significant jump to a mean MCC of 0.39/0.44 (APO/HOLO) is seen by selecting the top one energy ranked model, EN1, and these increase to 0.51/0.59 using the best of top five energy-ranked models, EN5. Clustering further improves model selection, with the best of top five clustering representative models, CL top5, yielding mean MCCs of 0.54/0.62, accuracies of 87%/89%, sensitivities of 57%/62%, specificities of 94%/95%, and precisions of 69%/77%, for the APO/HOLO sets (see Table 1). Interestingly, the top ranked cluster model, CL top1, has a MCC of 0.40/0.44, which is only slightly better than the EN1 model.

**Table 1:** DNA-binding site prediction benchmarks.

Model	MCC*	Accuracy*	Sensitivity*	Specificity*	Precision*
APO CL Top1	0.40±0.20	0.83±0.07	0.46±0.18	0.91±0.05	0.55±0.20
HOLO CL Top1	0.44±0.30	0.84±0.09	0.50±0.26	0.92±0.06	0.59±0.29
APO CL Top5	0.54±0.13	0.87±0.05	0.57±0.13	0.94±0.04	0.69±0.17
HOLO CL Top5	0.62±0.13	0.89±0.05	0.62±0.15	0.95±0.04	0.77±0.15

*: Means and standard deviations are shown for predictions on the APO/HOLO sets.

**Figure 4:**
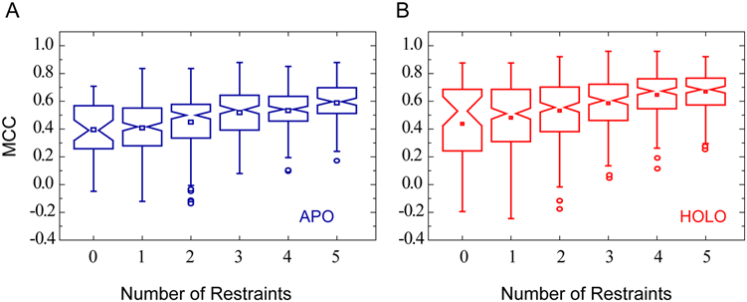
MCC of the Top1 clustering model *versus* number of geometric restraints applied for model filtration. In each case, up to five native DNA-binding residues were randomly selected as the restraint(s). Models in which the DNA does not contact these restraint residues were discarded, and the remaining models were subjected to clustering. Five independent trials were performed per restraint number per protein. Modeling was performed for both (A) APO and (B) HOLO sets.

Our method can readily take advantage of known information about DNA-binding sites, such as data collected from mutagenesis studies, NMR experiments, or sequence conservation analysis. The information can be used to derive contact restraints for model filtration [5],[7]. To illustrate this point, we randomly picked native DNA-binding protein residues and filtered all models in which these residues do not contact DNA. When applying more than one such restraint, we obtained significantly better top one models (Figure 4). The mean MCC values for the CL top1 models of apo-structures, for example, systematically increases from 0.40 without any restraint, to 0.45, 0.52, and 0.59 with two, three, and five restraints, respectively.

### DNA–Protein Interaction Mode

**Figure 5:**
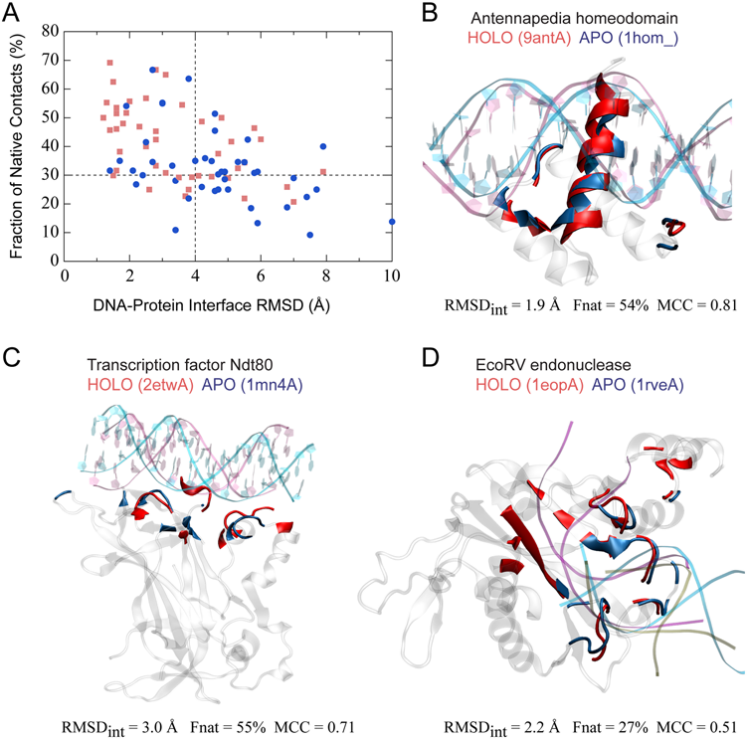
Native-likeness of predicted DNA-protein interaction modes. (A) RMSD of native DNA-protein interfacial residues *versus* the fraction of the native DNA-protein contacts observed in the model. The results of the best of the top five models are shown. The models are based on apo (blue circles) and holo (red square) protein structures, respectively. (B–D) Three examples illustrate the resemblance between the nonspecific DNA-protein complex model and the specific DNA-protein complex. All models are based on apo-structures. In each case, the model was superposed onto the native complex structure by optimally aligning the protein-DNA interfacial residues, colored in blue and red for the model and native protein structures, respectively. The transparent grey cartoons represent non-interfacial protein residues of the model. The nonspecific (dA·dT)_16_ B-DNA used for docking and the specific DNA fragment co-crystallized with the protein are colored in cyan and purple, respectively. In panel D, a non-cognate DNA from a third crystal structure is shown in brown. The DNA is placed such that the protein (PDB code 2revA, not shown) co-crystallized with the DNA is optimally aligned with its cognate native form. For clarity, only backbones are shown for the three DNAs in panel D. The PDB code includes the four-digit access code (lower case) and the chain identifier (upper case) of the protein. Graphic images were made with the program VMD [47].

Next, we compare interaction modes between representative nonspecific DNA-protein encounter complexes and the native (experimental) specific DNA-protein complexes. For this comparison, we need a mapping between the nonspecific DNA and the specific DNA complexed with the protein in the native structure. The mapping was obtained by gaplessly threading the nonspecific DNA along the native DNA with a scoring function that maximizes the overlap of the DNA-protein residue contacts. Then, the native DNA-protein contacts observed in the model were counted, and the RMSD of native interfacial residues relative to their positions in the model was calculated by optimally superposing these interfacial residues. For each protein, the best result of top five clustering models is shown in Figure 5A. In these models, the optimal alignment typically covers 85% of the length of the shorter DNA, and more than 95% of the native interfacial residues. On average, the fractions of native contacts (denoted as Fnat) observed in the model are 33%/41% for the APO/HOLO sets, respectively, and the corresponding DNA-protein interfacial RMSDs (denoted as RMSD_int_) are 4.6/3.4 Å. The results indicate some resemblance between nonspecific DNA-protein interaction modes and the specific-DNA-protein binding mode, though consistent specific base recognition cannot be expected due to the different DNA sequence employed and the possible conformational changes involved. About 70% of contacts involving specific base recognition in the specific complex are either lost or converted to backbone contacts in the corresponding nonspecific contacts.

From the prediction prospective, we may define a DNA-protein complex model as acceptable if the model satisfies one of the following two conditions: (i) Fnat≥30%, or (ii) Fnat≥10% and RMSD_int_≤4 Å, the criteria adopted from the Critical Assessment of PRedicted Interactions (CAPRI) [19]. Using these criteria, the predicted DNA-binding modes for 71%/86% of APO/HOLO proteins can be classified as acceptable, resulting in a mean RMSD_int_ of 3.9/3.1 Å and a mean Fnat of 37%/44%.

Three examples of predicted nonspecific DNA-protein complex models based on apo-structures are compared with the corresponding native specific DNA-protein complex structures in Figure 5B–D. The Antennapedia homeodomain from a *Drosophila melanogaster* transcription factor represents a classic DNA-binding domain that recognizes DNA through a helix-turn-helix motif [20],[21]. Using an apo protein structure [20], the best clustering model contains 14 DNA-interacting protein residues; all are among the 19 DNA-binding residues bound to the specific DNA sequence. The native-like binding mode of the predicted model is reflected by a RMSD_int_ of 1.9 Å and a Fnat of 54% (Figure 5B). The model, promoted from the sixth place on the energy ranking list to the second place through clustering, is the closest to the native structure among all 2500 docking solutions.

The second example from *Saccharomyces cerevisiae* Ndt80 is a DNA-binding domain belonging to the immunoglobulin-fold family of transcription factors [22],[23] (Figure 5C). The native DNA-protein interface exhibits a unique binding mode involving mainly loop residues. The top energy-ranked model correlates well with the native structure, having a MCC of 0.71, which is only slightly lower than the best value of 0.72 found among all docking solutions. The interfacial RMSD of 3.0 Å and Fnat of 55% suggest close similarity between the predicted and native binding mode.

The third example is a type II restriction endonuclease, EcoRV (Figure 5D), whose structures have been solved in DNA-free [24] and DNA-bound forms with either a cognate or a non-cognate DNA sequence [24],[25]. In the top energy-ranked model obtained with the unbound structure, residues involving DNA-protein interactions include about half of the protein residues contacting the cognate sequence in the experimental structure, yielding a moderate MCC of 0.51. The result is expected since the cognate DNA significantly deviates from the canonical B-DNA form by a bending angle of ∼50°, as shown in the native complex structure. As a result, the nonspecific DNA can only be partially aligned to the cognate DNA. In fact, the interaction mode presented by our model more closely resembles the binding mode of the non-cognate DNA-protein complex structure (Figure 5D). All ten DNA-binding residues involving non-cognate DNA recognition are predicted as DNA-binding according to our model. Note that EcoRV functions as a homodimer, and only the monomer was employed for docking.

### Application to Predicted Protein Models

Our approach was further validated on predicted protein models. First, the sequences of these 44 DNA-binding proteins were input into the threading algorithm PROSPECTOR_3.0 [26]. Depending on the confidence levels of the structural templates identified, proteins were classified into two groups: 30 Easy targets, which typically have good quality templates, and 14 Hard targets, which usually do not have a reliable template hit. Note that we excluded from the template library any structure that shares>30% global sequence identity with a given target. The best template, ranked by the TM-score structural similarity metric [27], has a mean RMSD of 7.9 Å with respect to the native holo-structure over about 92% alignment coverage, and the mean sequence identity of these templates is 19%. After TASSER runs for model assembly and refinement [16], the mean RMSDs of the top TASSER model and of the best of top five models were improved to 6.9 Å and 6.4 Å over the regions aligned with the templates. Overall, the mean TM-scores of the top and the best of five top models compared against the native holo structure are 0.61 and 0.63; the latter is ∼9% higher than the average TM-score of the best threading templates. Systematic model improvement over the best templates is evident, as an improved structural model was obtained in 37 of 44 cases.

**Figure 6:**
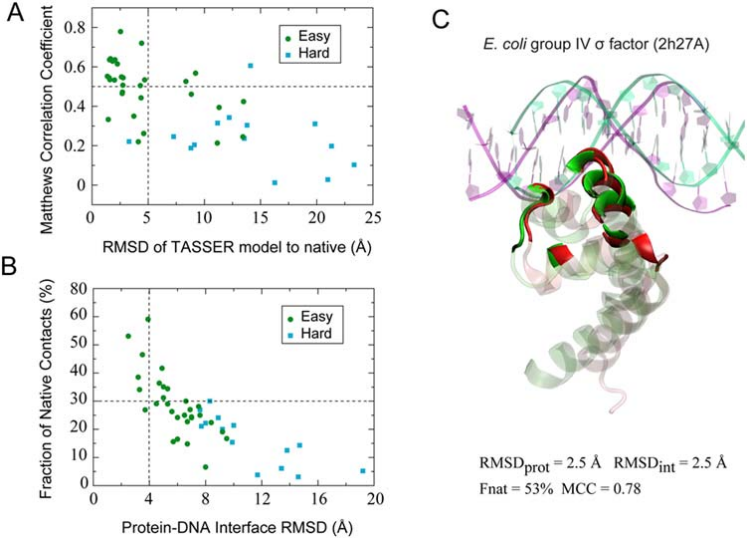
Prediction of DNA-protein binding interactions with TASSER models. (A) RMSD of the top TASSER model used for docking *versus* the MCC of DNA-binding site prediction. (B) RMSD to native of the DNA-protein interfacial residues *versus* the fraction of the native DNA-protein contacts observed in the model. (C) Example of a prediction with a TASSER model. The model (green) was superposed onto the native structure (red) by optimally aligning the DNA-protein interface. Interfacial protein residues observed in the TASSER model and native complexes are shown in solid colors, while non-interfacial protein residues are transparent. The nonspecific DNA used for docking and native DNA fragments co-crystallized with the protein are shown in green and purple ribbon representations, respectively.

For reach protein, the top TASSER model was employed for docking and subsequent analysis. The number of proteins whose top TASSER model has a RMSD≤5.0 Å from the native holo-structures is 24 (55%); all but one are from the easy set (Figure 6A). Among these 24 proteins, the best of top five DNA-protein complex models yields an average MCC/accuracy of 0.51/84% for DNA-binding site prediction. For the Easy/Hard sets, the best of top five models gives mean MCC of 0.50/0.23, a RMSD_int_ of 5.9/11.2 Å, and Fnat of 29%/16%, respectively (Figure 6B). While we obtained acceptable binding mode predictions for 12 (40%) of the targets from the Easy set, the predicted binding mode for Hard targets is generally incorrect, which is expected due to poor protein model quality. Overall, the binding site and mode predictions are satisfactory for the Easy set.

One example, the DNA-binding domain from an *E. coli* group IV σ factor, is illustrated in Figure 6C. The protein initiates transcription by binding to a specific promoter region and recruiting an RNA polymerase [28]. The closest template, an *Aquifex aeolicus* group I σ factor structure resolved in its DNA-free form, shares a sequence identity of 24% with the target. The top ranked TASSER model has an RMSD of 2.5 Å from the crystal protein structure (Figure 6C). The high quality model permitted us to build reliable docking complex models. The best of top five docking models predicts 11 of 15 DNA-binding amino acids at 92% precision; and the predicted interaction mode closely mimics the native binding mode exhibited by the crystal structure with an interfacial RMSD of 2.5 Å and Fnat of 53%.

### Comparison with Other DNA–Protein Pair Potentials

In addition to the DNA-protein energy function described above, we also tested the performance of three other statistical pair potentials proposed previously, including two quasichemical potentials, one at the residue, QC_Res_ [5] and two others at the all-atom level, QC_AA_ [29] and RAPDF [30] (see Methods). While the residue-level quasichemical potential uses a single distance cutoff of 4.5 Å, the all-atom potentials are distance dependent up to 10 Å. Since in previous studies, the potentials were derived from relatively small data sets, we re-parameterized these three potentials with the same set of 179 crystal complex structures used for our functional-group level quasichemical potential derivation [11]. Then, for each target from the APO/HOLO sets, the top 2500 docking solutions described above were re-ranked according to the energies calculated with the new potentials. Table 2 shows the results of binding site and mode predictions for the best of the top five models. On average, our energy function outperforms these three potentials. The mean MCC for the binding site prediction is 0.59/0.51 for the APO/HOLO sets using our energy function without clustering, compared with 0.55/0.47, from both the residue and all-atom quasichemical potentials, and 0.40/0.24 from the conditional probability scoring function RAPDF. Correspondingly, our energy function selected acceptable binding complex models in 77%/59% of the cases, whereas the residue-based and the two all-atom potentials selected acceptable models in 71%/50%, 71%/55%, and 40%/32% of the cases, respectively. These results suggest that detailed all-atom representations do not necessarily have an advantage over simplified residue or functional-group level potentials when applied to rank docking solutions from a non-specific DNA sequence. We also note that the clustering models, which have a mean MCC of 0.62/0.54, are significantly better than models selected by the three potentials (Wilcoxon signed-rank tests *P*<0.04).

**Table 2:** Comparison of DNA-protein pair potentials for model selection.

Ranking Schemes*	HOLO		APO	
	MCC†	N‡	MCC†	N‡
CL	0.62±0.13	38 (86%)	0.54±0.13	31 (71%)
EN	0.59±0.20	34 (77%)	0.51±0.16	26 (59%)
QC_Res_ †	0.55±0.19	31 (71%)	0.47±0.22	22 (50%)
QC_AA_ ‡	0.55±0.17	31 (71%)	0.47±0.17	24 (55%)
RAPDF^§^	0.40±0.37	21 (48%)	0.24±0.30	14 (32%)

*: CL and EN denote two scoring schemes using our DNA-protein interfacial energy function with and without clustering, respectively. QC_Res_ and QC_AA_ designate schemes using quasichemical pair potentials at the residue level with a single distance cutoff and at the all-atom level with multiple distance bins, respectively. The RAPDF scheme uses a scoring function proposed by Robertson and Varani [30]. For each scheme, the results of the best of top five ranked models are shown for the predictions on the APO/HOLO sets.

**†:** Mean and standard deviation of MCC are shown for the binding site predictions.

**‡:** The number (percentage) of proteins for which the predicted complex model are acceptable according to the CAPRI criteria.

### Comparison with Other DNA-Binding Site Prediction Methods

Our approach was compared with three established methods [9],[13],[14] that predict DNA-binding sites based on protein structures. Note that none of these three methods is capable of predicting the DNA-protein interaction mode. For the purpose of comparison, all calculations were carried out on the same set, AS62 [9], composed of DNA-binding protein structures in their holo-forms. As shown in Table 3, the top model from our approach already yields better results than previous methods on average. The mean MCC of our top model is 0.53, compared to 0.49 obtained independently by the Kuznetsov group [13] and by Tjong and Zhou's method named DISPLAR [14]. Moreover, the best of our top five models significantly improves the DNA-binding site prediction with a mean MCC of 0.62 and a mean accuracy of 87%, leading the results from the Kuzentsov method or DISPLAR by about one standard deviation unit. The latter two methods perform better than that proposed by Ahmad *et al.* [9]. This reason can be partially attributed to the fact that the Ahmad *et al.* did not use position-specific sequence profiles in their method.

**Table 3:** Comparison of DNA-binding site prediction methods.

Method	MCC*	Accuracy*	Sensitivity*	Specificity*	Precision*
CL Top1	0.53±0.20	0.85±0.07	0.59±0.20	0.93±0.05	0.68±0.19
CL Top5	0.62±0.14	0.87±0.06	0.66±0.16	0.94±0.05	0.75±0.15
Kuznetsov *et al.* †	0.49±0.17	0.78±0.08	0.79±0.15	0.77±0.10	( 0.43 )
Tjong and Zhou‡	0.49±0.19	0.81±0.09	0.67±0.29	0.83±0.13	0.57±0.22
Ahmad *et al.* §	—	0.79	0.40	0.82	—

*: Mean and standard deviation are shown for the predictions on the AS62 set, except as otherwise noted.

**†:** Data taken from reference [13], except the mean precision shown in the parentheses. The mean precision, not given in the original reference, was estimated by using the values of mean sensitivity and specificity, and a DNA-binding residue faction of 0.18.

**‡:** Predictions made by DISPLAR [14] web server at http://pipe.scs.fsu.edu/displar.html, and the measures were calculated as described in the Methods.

**§:** Data taken from reference [9], where only the means were provided.

We further compared the performance of our method on apo structures with DISPLAR. The predictions of DNA-binding sites of 44 proteins structures from the APO set were performed using the DISPLAR webserver. The averages of MCC/accuracy by DISPLAR are 0.39/82.5%, which are slightly lower than 0.40/82.7% from the results by the first ranked model of our method. The difference is statistically insignificant. However, the performance of the best of the top five models by our method, 0.54/86.7%, is significantly better than that of DISPLAR (Wilcoxon signed-rank test *P*<0.001). In practice, the multiple (but limited number of) models generated by our method can be filtered through incorporation of existing experimental studies on binding-sites, thereby further improving the prediction.

### Effects of Conformational Changes

The difference between the predicted docking model and the native complex structure may be explained by two main reasons: First, nonspecific instead of specific DNA was used for docking. Second, rigid-body docking does not consider the conformational changes of either the DNA or the protein. The effects of conformational changes in protein are clear as holo-structures consistently produce models closer to the native state than those using apo-structures. In principle, by also taking DNA conformational changes into account, one should be able to obtain improved models.

**Figure 7:**
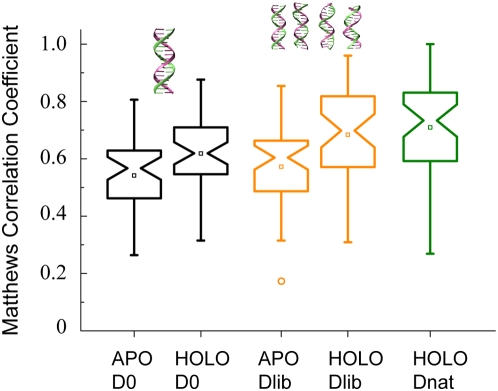
Improvement on DNA-binding site prediction using a DNA library composed of poly dA·dT DNA in various conformations. D0 denotes the canonical B-DNA described above. Dlib denotes a library of four 16 bp dA·dT B-DNA structures, which are shown in the cartoon representations. Dnat denotes the 44 native-like poly dA·dT DNA structures, each of which was built by keeping the original sugar-phosphate backbone of the native DNA structure from the holo-form complex and mutating the specific base pairs into the dA·dT base pair using the base step geometry parameters of the native DNA. The results shown are from the best of top five clustering models.

The flexibility problem can be partially addressed through docking the protein to a library of DNA in various conformations [7]. To explore this idea, we constructed a DNA library composed of three poly dA·dT B-DNA structures, whose backbone RMSDs range from 1 to 3 Å with respect to the canonical B-DNA used above, and the canonical B-DNA itself (see Table S2). For convenience, we name the canonical B-DNA as D0, and the DNA library as Dlib. Using Dlib, we obtained complex models generated by docking the protein to each DNA in the library. For each of the four protein-DNA combinations, the same docking procedure described above was followed, and the top five clustering models were selected and pooled together. From this pool of twenty clustering models we selected top five models according to their interfacial energy. As shown in Figure 7, the mean MCCs of DNA-binding residues predictions are improved from 0.54/0.62 (D0 docking) to 0.57/0.68 (Dlib docking) for the APO/HOLO sets, respectively.

One can further estimate the upper limit of such improvement by docking holo protein structures to nonspecific DNA that adopts the native specific-DNA conformation, though in general one cannot assume that the nonspecific DNA associates with the protein in exactly the same conformation as the specific DNA. In this estimation, we took the native DNA structures from the 44 complex structures and mutated all base pairs into dA·dT with the program 3DNA [31]. We name this set of DNA structures Dnat. Each protein structure from the HOLO set was then docked to the corresponding DNA structure in Dnat. The resulting average MCC for binding site prediction from the best of top five clustering models is 0.71 (Figure 7), which is slightly higher than 0.68 from docking holo protein forms to Dlib. While we expect to see further improvement with fully flexible docking, it poses a challenging problem in practice [7],[32]. So far, successful examples are limited to local refinement, which requires that the initial rigid body models subjected to flexible refinement are sufficiently close to their native conformation. A thorough study on flexible docking, however, is beyond the scope of the current study.

## Discussion

How a DNA-binding protein locates its specific DNA target sequence is a fundamental, unsolved problem in biology. It has been proposed that association with nonspecific DNA sequences and subsequent travel along the sequence facilitates the search for the specific DNA target sequence [17],[18]. In this regard, it has been shown that specific DNA-binding proteins, such as transcription factors and restriction endonucleases, can locate target sites at rates several orders of magnitude faster than that estimated by random three-dimensional diffusion, through mechanisms known collectively as facilitated diffusion [17],[18]. A crucial step of the facilitated diffusion processes involves the association of the protein with a nonspecific DNA sequence; this is followed by one-dimensional sliding along the DNA or hopping over short distances to accelerate the search for a specific DNA target sequence. Despite recent advances that provide visualizations of protein sliding along DNA [33], the structural details of how a DNA-binding protein associates with a nonspecific DNA remain elusive, primarily due to weak interactions between nonspecific DNA and the protein. Indeed, due to the fact that the interactions are nonspecific, there exist only a few solved atomic structures for nonspecific DNA-protein complexes [24],[34]. Our study provides useful structural insights into how a specific DNA-binding protein interacts with a nonspecific DNA sequence during the facilitated diffusion process. The similarity between the specific DNA-protein interaction mode and nonspecific interaction modes may reflect an important sampling step in search of its specific DNA targets by a DNA-binding protein.

By systematically studying encounter complexes of 44 specific DNA-binding proteins with a nonspecific DNA molecule, we found that the vast majority of these DNA-binding proteins favorably interact with nonspecific DNA at the same binding sites for their specific DNA targets. Using APO/HOLO-structures for docking and a pair potential for energy ranking, we obtained at least one near-native model among the top ten models for 77%/84% of APO/HOLO proteins. In these models, protein residues that contact the nonspecific DNA coincide with those that contact the specific DNA with a MCC>0.5. By introducing a clustering procedure, the most native-like model among the top five cluster representatives has an average MCC of 0.54/0.62 when APO/HOLO structures are used. Moreover, the DNA-protein interaction modes observed in these models resemble the corresponding native binding modes with specific DNA. The average interfacial RMSD is 4.6/3.4 Å, and the fraction of native contacts observed is 33%/41% for APO/HOLO proteins, respectively.

Our results therefore suggest that a DNA-binding protein frequently samples nonspecific DNA using the same binding sites as used for specific DNA recognition. The results are consistent with a recent Langevin dynamics study on the diffusion of three DNA-binding proteins along nonspecific DNA [35], and are also consistent with the few available atomic structures of DNA-binding proteins in complex with both specific and nonspecific DNA [24],[34]. One interesting example is the endonuclease EcoRV, which locates a specific cleavage site through a combination of 1D sliding along nonspecific sequence and 3D jumping [36],[37]. The nonspecific DNA recognition observed in our top model and in a crystal structure of the nonspecific DNA-EcoRV complex involves the same set of protein residues which also participate in specific DNA recognition [24]. However, the majority of native contacts formed in the cognate DNA-protein complex structure are lost in our model, largely due to the absence of the dramatic bending exhibited by the cognate DNA.

The overlap of nonspecific and specific DNA interaction sites on the protein surface allows us to predict DNA-binding residues. The best of top five models generated with holo-structures have an average MCC of 0.62, which is 15% higher than the average MCC of 0.54 obtained with apo-structures. Despite the notable difference, the performance of our method is satisfactory for apo-structures. This validation on apo-structures has important practical applications. Going beyond the DNA-binding site prediction, our method also provides models for the DNA-protein interaction modes. For 86%/71% of HOLO/APO structures, at least one of the top five models exhibits an interaction mode somewhat similar to the native binding mode, with a mean RMSD_int_ of 3.1/3.9 Å and a Fnat of 44%/37%. These complex models are acceptable using CAPRI criteria [19].

The performance of our method in DNA-binding site prediction has been compared with three machine-learning based methods. We note that the top model by our method already performs better than the other methods in terms of MCC and overall accuracy. While machine learning based methods typically provide only one model for assessment, our method generates a limited number of representative models for selection. This can be a great advantage for practical application, since incorporation of existing experimental studies on binding-sites may greatly improve model selection. On average, the best of our top five models by our method achieves a MCC of 0.62 and accuracy of 87%, which is significantly better than the MCC of 0.49 and accuracy of 81% of DISPLAR [14], the best among other methods. In addition, our method has the advantage of predicting the binding mode, an ability that the machine-learning methods lack. A downside of our method, however, is that it is computationally more demanding than machine-learning methods, typically requiring hours *versus* minutes of computation time for one target. Nevertheless, given the widespread availability of computational resources, this is not a significant limitation.

Despite these successes, the method is not designed for predicting the specific DNA sequence recognized by a DNA-binding protein; this is a related, yet very challenging problem. Knowledge-based distance-dependent contact potentials at the residue [4] or the all-atom level [29],[30],[38], and physics-based all-atom potentials [39],[40], have been applied to predict DNA specificity. While these studies have reported success on a few cases, they are limited to known atomic complex structures or models from closely related complex structures with almost identical DNA-binding interface. Nevertheless, they suggest that a successful approach must address structural flexibility and cooperativity among partners that form a DNA-protein complex.

Another interesting question is whether one can use the current approach to determine DNA-binding function given a protein structure. To explore this issue, we applied the method to ∼3,000 non-DNA-binding proteins collected previously [11]. Unfortunately, we were not able to derive a practical interfacial energy threshold to differentiate DNA-binding proteins from non-DNA-binding proteins, despite the notable difference of average interfacial energy. For DNA-binding function prediction, the knowledge based approach DBD-Hunter [11], which requires that the structure of a target protein be related to that of a known DNA binding protein, seems more appropriate. Future efforts may involve expanding the template library for DBD-Hunter by adding complex structure models obtained from the current approach.

In the post-genomic era, the rapid progress of structural genomics projects has greatly advanced our knowledge about structural biology. Each year thousands of new protein structures have been determined and deposited to the PDB. In principle, the accumulation of protein structures enables a practical solution to the folding problem through template based modeling [16]. Using the well-established modeling method, TASSER, we have obtained a top ranked protein model within 5 Å from their native structures for over half of the 44 DNA-binding proteins. These models were constructed and refined from homologous/analogues templates with less than 30% sequence identity. We have demonstrated that one can satisfactorily predict DNA-binding sites using these good models. The average MCC and accuracy are 0.51 and 84% for the best of top five complex models. This is roughly comparable to the performance when experimentally solved apo-structures are used. Ultimately, the combination of modeling and DNA-protein docking may lead the way to the high throughput prediction of DNA-protein interactions.

## Methods

### Data Sets

#### APO/HOLO sets

A total of 44 pairs of DNA-binding protein structures determined both in the DNA-bound (HOLO) and unbound (APO) forms were selected from a previous study [11] using the following criteria: (i) the holo- and apo-structures share>90% global sequence identity; (ii) the protein is bound to a specific DNA molecule in the holo-form; (iii) the protein chain length is less than 400 residues; and (iv) the DNA bound to protein has more than 7 and less than 40 base pairs. These proteins include 29 transcription factors, 12 enzymes, and 3 other types of DNA-binding proteins (Table S1). All share<35% global sequence identity among each other.

#### AS62 set

For comparison to other DNA-binding site prediction methods, we adopted a widely used set of 62 DNA-protein complex structures [9]. To reduce redundancy, we followed Ref. [13] and removed identical protein chains from these structures, resulting in 66 protein chains for the benchmark test.

### Protein–DNA Complex Modeling

A flowchart of the modeling protocol is provided in Figure 1. In the first step, a DNA-binding protein was docked to a poly(dA·dT)_16_ B-DNA with the FFT-based rigid-body docking program FTDOCK [5]. A grid size of 0.7 Å, a rotation angle step of 12°, and surface thickness of 1.2 Å were employed for docking. The B-DNA structure was built with the program 3DNA [31], using a canonical B-DNA fiber model. The top 10,000 docking models ranked by the shape complementarity score were retained. These models were subsequently filtered by the requirement that the protein must contact at least one heavy atom from the two central DNA base pairs. This helps to reduce the redundancy of the models due to the helical symmetry of the DNA and also to remove models in which the protein clashes with DNA termini. The remaining complex models were re-ranked according to their DNA-protein interfacial energy given by
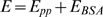
(1)where 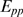 is a statistical pair potential at the functional group level [11], and 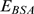 is a surface burial term given by −0.02 kT/Å^2^ × Buried Surface Area (BSA). BSA was calculated with the program NACCESS [41]. The statistical pair potential was developed from an analysis in 179 DNA-protein complex structures [11]. For each target, we derive a corresponding potential by excluding any homologous protein with >35% sequence identity from the 179 complex set and repeat the analysis. The top 2500 energy-ranked models were retained for clustering, which uses the coordinates of the COM of DNA-binding protein residues. The clustering procedure starts by selecting the top energy-ranked model as a clustering seed. All models within a COM distance of 6 Å from the seed are assigned to this cluster, and removed from subsequent clustering. We then repeat this procedure until no model is left. Finally, the clusters were ranked using the average energy of all members in each cluster. From each cluster, we select the lowest energy model as the representative model.

### Model Assessment

A protein residue is assigned to be DNA-binding (or DNA-interacting) if at least one heavy atom from the protein residue is within 4.5 Å of at least one heavy atom from the DNA. Using this definition, about 18% of protein residues can classified as true DNA-binding in the analysis of the HOLO set. Given the imbalanced nature of the DNA-binding residues and non-DNA-binding residues, the Matthews correlation coefficient is a suitable metric for assessing overlap or prediction of DNA-binding residues between an encounter complex and the native complex. The MCC is defined by [42]

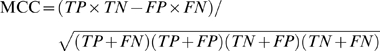
where TP, FP, TN, and FN are true positives, false positives, true negatives and false negatives, respectively. A true positive refers to a DNA-binding protein residue observed in the native specific complex. Other performance measures calculated are the following:
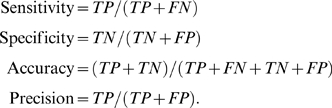



In the DNA-binding mode analysis, we mapped the nonspecific DNA to the specific DNA by maximizing DNA-protein contact overlap. A DNA-protein contact is defined at the residue level. The RMSD between two structures was calculated using the coordinates of backbone Cα and/or DNA C1′ atoms. The interfacial RMSD was calculated for interfacial protein/DNA residues observed in the native specific-DNA-protein complex structure.

### Protein Structure Modeling

The structures of the 44 proteins from the APO/HOLO sets were predicted following the TASSER methodology [16]. Briefly, a target sequence was threaded against a non-redundant protein structure library by the program PROSPECTOR_3 [26], and the resulting structure templates are used for subsequent model assembly and refinement by the program TASSER, which uses a Monte Carlo replica exchange algorithm for sampling. Note that we excluded any template that shares>30% global sequence identity with the target. The replica trajectories were clustered and representative models generated from these clusters. We built all-atom protein models from the reduced-atom TASSER models with the program PULCHRA [43]. In this study, the top ranked TASSER model is employed for DNA-docking.

### Statistical Pair Potentials

Four knowledge-based statistical DNA-protein pair potentials were developed from an analysis of 179 non-redundant DNA-protein complex crystal structures [11]. These include three quasichemical potentials at the residue [5], functional-group [11], and all-atom [29] levels, and another all-atom potential (termed RAPDF, residue-specific all-atom conditional probability discriminatory function) using a different reference state [30]. RAPDF was originally derived using the Bayesian probability formalism [30],[44]; it can be expressed equivalently under the Boltzmann distribution formalism. Here, we introduce all these potentials using the Boltzmann formalism, which assumes that the frequencies of observed pair interaction states follow a Boltzmann distribution [45]. Consequently, the pair interaction energy *E* can be deduced from the inverse of Boltzmann's law
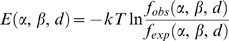
(2)where α and β are protein/DNA residues, functional-group, or heavy-atom types for the corresponding potentials, respectively, and, 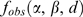 and 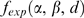 are the observed and expected frequencies of the αβ pair at the distance *d*, respectively. For residue and functional-group level potentials, the distance *d* is defined as the minimum distance between a pair of heavy atoms from the corresponding the αβ pair; and a single distance cutoff of 4.5 Å was used. Multiple distance bins from 3 Å to 10 Å with a bin width of 1 Å were employed for the two all-atom potentials. The observed frequency can be obtained by
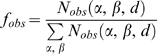
(3)where 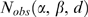 denotes the number of observed αβ contact pairs at the distance *d*. For quasichemical potentials, the expected frequency is given by
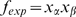
(4)where 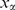 and 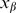 are the mole fractions of type α and β. The mole fraction for each type is the overall mole fraction in the entire template library, following a scheme known as the composition-independent scale [46]. For RAPDF, the expected frequency is estimated by
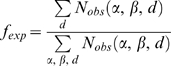
(5)


For a DNA-protein complex structure, the corresponding DNA-protein interfacial energy is the summation of all observed pair interactions in the structure.

The RAPDF parameterization was performed using the program implemented previously [30]. In a benchmark test on the DNA-protein docking decoy set compiled by Robertson and Varani [30], our new set of RAPDF parameters yield an average Z-score of −11.0 for the native complex structures, slightly better than the previous average Z-score of −9.6 obtained by parameters determined on a smaller set composed of 52 DNA-protein complex structures.

### Availability

A web-server implementation of the method described here is available at http://cssb.biology.gatech.edu/skolnick/webservice/DP-dock/.

## Supporting Information

Table S1 List of the DNA-binding proteins in the APO/HOLO sets
(0.10 MB DOC)

Table S2 List of four B-DNA structures used in the DNA library
(0.06 MB DOC)
