# Supplementary material for: From Nonspecific DNA–Protein Encounter Complexes to the Prediction of DNA–Protein Interactions
Source: PLoS Comput Biol. 2009 Apr 3;5(3):e1000341. doi: 10.1371/journal.pcbi.1000341 (PMC2659451; doi:10.1371/journal.pcbi.1000341)
Supplement: Table S1 — List of the DNA-binding proteins in the APO/HOLO sets (0.10 MB DOC) [file pcbi.1000341.s001.doc]

**Table S1**. List of the DNA-binding protein sets APO/HOLO*.

| **APO** | **HOLO** | **Residue Range** | | **Group** | **Protein Description** |
| --- | --- | --- | --- | --- | --- |
| 1a41_ | 2h7gX |  | EZ | | Type IB Topoisomerase |
| 1af5_ | 1n3fA |  | EZ | | Endonuclease I-CreI |
| 1ajyA | 1zmeC |  | TF | | PUT3 |
| 1arrA | 1parA |  | TF | | Arc repressor |
| 1aw6_ | 1d66A |  | TF | | GAL4 |
| 1es8A | 1dfmA |  | EZ | | Endonuclease BglII |
| 1ev7A | 1iawA | 177-309 | EZ | | Endonuclease NaeI |
| 1evxA | 1cz0A |  | EZ | | Endonuclease I-PpoI |
| 1f9fA | 1jj4A |  | EZ | | Papillomavirus E2 |
| 1fc3A | 1lq1A |  | TF | | Spo0A |
| 1g6nA | 1zreA | 138-206 | TF | | Catabolite gene activator |
| 1gv2A | 1h8aC | 89-143 | TF | | c-Myb |
| 1gvjA | 1k7aA | 333-436 | TF | | ETS-1 |
| 1gxqA | 1gxpA |  | TF | | PhoB |
| 1h56A | 1f0oA |  | EZ | | Endonuclease PvuII |
| 1hom_ | 9antA | 5-60 | TF | | Antennapedia homeodomain |
| 1iknA | 1vkxA | 19-191 | TF | | NF-kappa B |
| 1irqA | 2bnwA |  | TF | | Repressor omega |
| 1j0rA | 1f4kA |  | OT | | Replication terminator protein |
| 1jbgA | 1r8dA |  | TF | | MerR |
| 1jtxB | 1jt0A | 2-72 | TF | | QacR |
| 1mjkA | 1mjmA |  | TF | | MetJ |
| 1mn4A | 2etwA |  | TF | | Ndt80 |
| 1okrA | 1saxA |  | TF | | Methicillin repressor MecI |
| 1or7A | 2h27A | 123-187 | TF | | Group IV sigma factor |
| 1pra_ | 1rpeL | 1-63 | TF | | bacteriophage 434 repressor |
| 1pyc_ | 1hwtC | 60-97 | TF | | Hap1 |
| 1r05A | 1nlwB |  | TF | | Max |
| 1rveA | 1eopA |  | EZ | | Endonuclease EcoRV |
| 1rxr_ | 1by4A |  | TF | | Retinoid X receptor |
| 1sdoA | 1vrrA | 1-203 | EZ | | Endonuclease BstYI |
| 1tfb_ | 1c9bA | 111-207 | TF | | Transcription factor IIB |
| 1vf9A | 1w0uA | 446-500 | OT | | Telomeric protein TRF2 |
| 1vhiA | 1b3tA |  | OT | | Epstein-Barr nuclear antigen 1 |
| 1vokA | 1qnbA | 16-115 | TF | | TATA box-binding protein |
| 1wpkA | 1u8bA | 9-76 | EZ | | Ada |
| 1wtdA | 1wteA |  | EZ | | Endonuclease Eco0109I |
| 1xwrA | 1zs4A |  | TF | | Lambda cII |
| 1z91A | 1z9cA |  | TF | | OhrR |
| 2audA | 1tx3A |  | EZ | | Endonuclease HincII |
| 2cpgA | 1b01A |  | TF | | CopG |
| 2fejA | 2ahiA |  | TF | | p53 tumor suppressor |
| 2jcgA | 1rzrG | 3-60 | TF | | Catabolite Control Protein A |
| 2tdx_ | 1ddnA |  | TF | | Diphtheria toxin repressor |

* Each entry is provided with the four-digit PDB codes and the protein chain identifiers for both APO/HOLO protein structures, the residue range of the DNA-binding domain if not the whole chain, the group name, and the description of the protein. Proteins are classified into three groups: transcription factor (TF), enzyme (EZ), and the others (OT).
