# Supplementary material for: From Nonspecific DNA–Protein Encounter Complexes to the Prediction of DNA–Protein Interactions
Source: PLoS Comput Biol. 2009 Apr 3;5(3):e1000341. doi: 10.1371/journal.pcbi.1000341 (PMC2659451; doi:10.1371/journal.pcbi.1000341)
Supplement: Table S2 — List of four B-DNA structures used in the DNA library (0.06 MB DOC) [file pcbi.1000341.s002.doc]

**Table S2.** List of four B-DNA structures used for building the DNA library*.

| **Name** | **D0** | **D1** | **D2** | **D3** |
| --- | --- | --- | --- | --- |
| **PDB** | − | 1hwt | 1gxp | 1z9c |
| **Chain** | − | A:5-20  B:1-20 | C:2-17  D:7-22 | G:5-20  H:10-25 |
| **RMSD (Å)** | 0 | 1.3 | 1.9 | 3.0 |
| **Shift (Å)** | 0.00 | -0.05 (0.96) | 0.01 (0.36) | -0.03 (0.43) |
| **Slide (Å)** | 0.45 | 0.20 (0.80) | -0.17 (0.89) | -0.32 (0.46) |
| **Rise (Å)** | 3.36 | 3.33 (0.16) | 3.41 (0.26) | 3.28 (0.12) |
| **Tilt (°)** | 0.00 | 0.11 (2.58) | 0.26 (3.98) | 0.21 (2.25) |
| **Roll (°)** | 1.71 | 0.40 (4.12) | 2.60 (3.50) | 5.01 (4.44) |
| **Twist (°)** | 35.96 | 37.13 (5.79) | 33.84 (2.78) | 33.28 (3.88) |
| **X-disp (Å)** | 0.49 | 0.15 (1.47) | -0.79 (1.46) | -1.38 (1.03) |
| **Y-disp (Å)** | 0.01 | 0.06 (1.35) | 0.09 (0.92) | 0.11 (0.78) |
| **h-Rise (Å)** | 3.38 | 3.34 (0.21) | 3.37 (0.28) | 3.18 (0.20) |
| **Inclination (°)** | 2.76 | 0.81 (6.52) | 4.53 (6.15) | 8.50 (7.92) |
| **Tip (°)** | 0.00 | -0.22 (3.96) | -0.69 (6.54) | -0.30 (3.94) |
| **h-Twist (°)** | 36.00 | 37.42 (5.79) | 34.31 (2.77) | 33.98 (3.85) |

* Each DNA structure is provided with the PDB code, chain identifiers, and the corresponding residue range, with the exception of the canonical B-DNA D0, which was taken from [1]. Backbone RMSDs of D1 to D3 with respect to D0 are calculated. The means (standard deviations) of local base pair parameters are calculated with 3DNA [2]. The exact definitions of these parameters can be found in [2]. To construct the poly dA·dT library, all base pairs of D0 to D3 were mutated with the program 3DNA [2] to dA·dT using the base step geometry parameters of the native DNA.

## Supplementary References

1. Arnott S (1999) Polynucleotide secondary structures: an historical perspective. In: Neidle S, editor. Oxford Handbook of Nucleic Acid Structure. Oxford, UK: Oxford University Press. pp. 1-38.

2. Lu XJ, Olson WK (2003) 3DNA: a software package for the analysis, rebuilding and visualization of three-dimensional nucleic acid structures. Nucleic Acids Research 31: 5108-5121.
